# *In Vivo* Acute on Chronic Ethanol Effects in Liver: A Mouse Model Exhibiting Exacerbated Injury, Altered Metabolic and Epigenetic Responses

**DOI:** 10.3390/biom5043280

**Published:** 2015-11-20

**Authors:** Shivendra D. Shukla, Annayya R. Aroor, Ricardo Restrepo, Kusum K. Kharbanda, Jamal A. Ibdah

**Affiliations:** 1 Department of Medical Pharmacology and Physiology, School of Medicine, University of Missouri, Columbia, MI 65212, USA; E-Mails: aroora@health.missouri.edu (A.R.A.); restreporj@health.missouri.edu (R.R.); 2 Veterans Affairs Nebraska-Western Iowa Health Care System, Omaha, NE 68105, USA; E-Mail: Kusum.Kharbanda@va.gov; 3 Department of Internal Medicine, School of Medicine, University of Missouri, Columbia, MI 65212, USA; E-Mail: ibdahj@health.missouri.edu

**Keywords:** acute ethanol, acute on chronic ethanol, alcoholic liver disease, binge alcohol, chronic-binge alcohol, epigenetics, histone modifications, mouse liver

## Abstract

Chronic alcoholics who also binge drink (*i.e.*, acute on chronic) are prone to an exacerbated liver injury but its mechanism is not understood. We therefore investigated the *in vivo* effects of chronic and binge ethanol ingestion and compared to chronic ethanol followed by three repeat binge ethanol on the liver of male C57/BL6 mice fed ethanol in liquid diet (4%) for four weeks followed by binge ethanol (intragastric administration, 3.5 g/kg body weight, three doses, 12h apart). Chronic followed by binge ethanol exacerbated fat accumulation, necrosis, decrease in hepatic SAM and SAM:SAH ratio, increase in adenosine levels, and elevated CYP2E1 levels. Histone H3 lysine acetylation (H3AcK9), dually modified phosphoacetylated histone H3 (H3AcK9/PS10), and phosphorylated H2AX increased after binge whereas phosphorylation of histone H3 ser 10 (H3S10) and H3 ser 28 (H3S28) increased after chronic ethanol-binge. Histone H3 lysine 4 and 9 dimethylation increased with a marked dimethylation in H3K9 in chronic ethanol binge group. Trimethylated histone H3 levels did not change. Nuclear levels of histone acetyl transferase GCN5 and histone deacetylase HDAC3 were elevated whereas phospho-CREB decreased in a distinctive manner. Taken together, acute on chronic ethanol ingestion caused amplification of liver injury and elicited characteristic profiles of histone modifications, metabolic alterations, and changes in nuclear protein levels. These findings demonstrate that chronic ethanol exposure renders liver more susceptible to repeat acute/binge ethanol induced acceleration of alcoholic liver disease.

## 1. Introduction

Binge drinking associated with chronic alcohol consumption is a major public health concern in the United States and around the globe [1,2,3,4]. Binge drinking in adolescent and young adult populations is rising at alarming proportions [5,6]. Most of the patients admitted to hospital with liver disease are alcoholics with binge drinking patterns [2,3,4]. Heavy binge drinking by chronic alcohol abusers is the trigger for augmenting liver injury [7,8,9] but the molecular mechanism sensitizing the progression of liver injury by binge after chronic ethanol intake is poorly understood.

Oxidative stress, increase in adenosine levels, and dysregulated methionine metabolism are considered to be contributing factors for the progression of liver injury [10,11,12,13] and the role of epigenetic mechanisms mediating progression of alcoholic liver injury is increasingly being recognized [14,15]. Among the epigenetic factors regulating gene expression, differential pattern of epigenetic histone modifications also determine activation of different sets of genes. Stable epigenetic histone modifications impact long term and transgenerational effects on gene expression but dynamic changes contribute to short term or prolonged effects on gene expression [16,17]. Epigenetic histone modifications are implicated in liver injury by binge [14,15,18,19,20,21,22,23,24], chronic alcohol consumption [25,26,27,28], and chronic alcohol administration followed by binge [29,30].

We have shown in a clinically relevant rat model that chronic ethanol followed by repeat ethanol binge is accompanied by augmentation of liver injury [30]. In the present study, we have utilized mice model to examine the effects and potential mechanisms underlying alcohol induced liver injury [31,32].

## 2. Results

### 2.1. Increased Hepatic Steatosis, Necrosis, Adenosine, CYP2E Levels and Decreased S-Adenosylmethionine by Binge Administration in Chronic Ethanol Treated Mice

**Figure 1:**
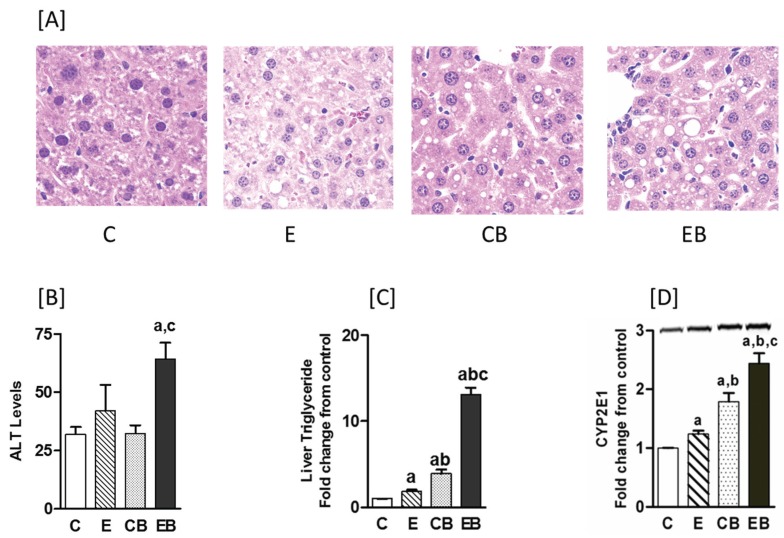
Hepatic steatosis, serum ALT, and CYP2E1 levels in chronic ethanol and binge treated mice. The chronic ethanol feeding (four weeks) and three repeat binge treatment was performed as described under “Experimental Section”. (**A**) Hepatic steatosis: sections of liver samples were stained with hematoxylin and eosin; (**B**) Serum ALT levels; (**C**) Liver triglyceride levels; (**D**) CYP2E1 protein measured by western blotting (representative blot images shown above the histogram). Values are mean ± SE (*n* = 3 to 4 mice). a: significant compared to control (*p* < 0.05); b: significant from chronic ethanol group (*p* < 0.05); c: significant compared to control-binge. C: Control (pair fed); E: Chronic ethanol; CB: Control ethanol binge; EB: Chronic-ethanol-binge.

Chronic ethanol administration or acute repeat binge caused only a moderate increase in liver steatosis, which was mainly microvascular, whereas there was a marked macrosteatosis in the chronic-binge ethanol group (Figure 1A). Liver necrosis, as determined by measurement of ALT levels, increased modestly above control in chronic ethanol but was statistically insignificant. In chronic ethanol followed by binge treatment, ALT increased significantly by 275% above control (Figure 1B). Triglyceride levels increased in all groups above control (Figure 1C). Compared to control group, the TG levels were 2, 4, and 13 folds higher in E, CB, and EB groups, respectively. Noticeably, the highest TG levels were in chronic ethanol-binge (EB) group that was about four fold higher than binge alone group (CB), and about seven fold higher than the chronic ethanol (E) group. Chronic ethanol increased CYP2E1 protein levels only modestly (Figure 1D) while binge ethanol generated a much higher CYP2E1 levels compared with controls. The largest increase (2.5 fold) was seen in the chronic ethanol-binge group (Figure 1D).

**Figure 2:**
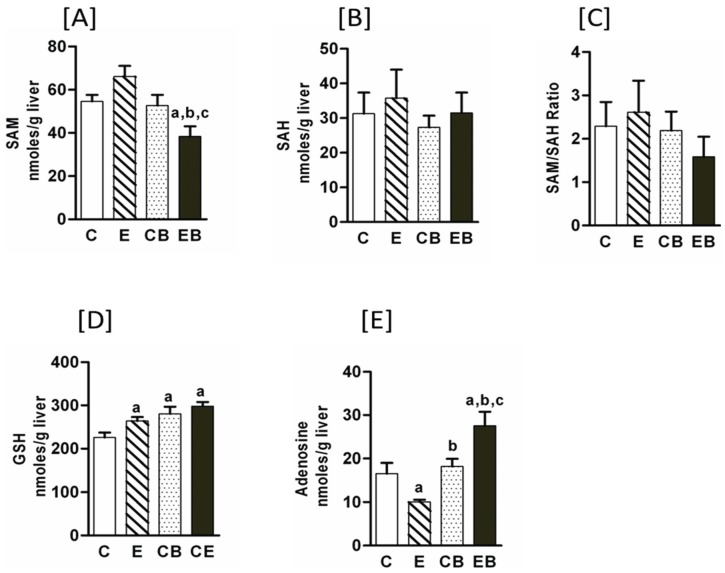
Hepatic *s*-adenosylmethionine (SAM), *s*-adenosyl homocysteine (SAH), adenosine and glutathione levels in chronic ethanol and binge treated mice. Experimental protocol was similar to that described in “Experimental Section”. Values are mean ± SE (*n* = 4 mice). a: significant compared to control (*p* < 0.05); b: significant from chronic ethanol group (*p* < 0.05); c: significant compared to control-binge. C: Control (pair fed); E: Chronic ethanol; CB: Control ethanol binge; EB: Chronic-ethanol-binge. (**A**) SAM; (**B**) SAH; (**C**) SAM/SAH ratio; (**D**) GSH; (**E**) Adenosine.

Dysregulated methionine metabolism has been reported in chronic ethanol treated mice [11,25]. Therefore, we determined hepatic levels of *s*-adenosyl methionine (SAM) and *s*-adenosyl homocysteine (SAH). Under the experimental conditions used in the present study, chronic ethanol increased SAM levels but a subsequent binge in chronic ethanol treated group resulted in a decrease that was statistically significant (Figure 2A). SAH levels were not significantly different among chronic ethanol, binge, and chronic ethanol binge groups. In chronic followed by three binge (EB) samples there was an apparent decrease in SAM/SAH ratio as compared to Control (C), chronic ethanol (E), or control binge (CB) groups (Figure 2C). Chronic ethanol increased GSH and binge on chronic had no further effect on GSH levels (Figure 2D). Upregulation of adenosine signaling has been shown to contribute to chronic alcoholic liver injury [12].

Hence, hepatic adenosine concentration in chronic, binge and chronic binge ethanol group were evaluated (Figure 2E). Hepatic adenosine levels significantly decreased in chronic ethanol treated mice, but their levels increased by binge ethanol with the highest levels in chronic ethanol-binge liver (Figure 2E).

### 2.2. Increased Phosphorylation of Histone H3 after Chronic Ethanol-Binge

**Figure 3:**
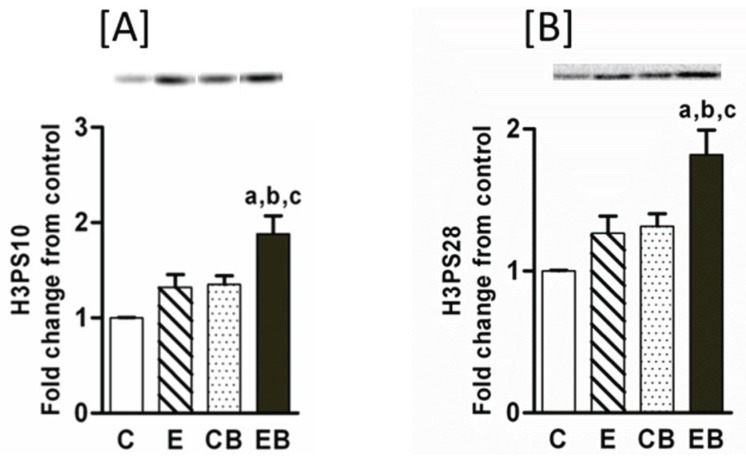
Phosphorylated histone H3S10 and S28 in chronic and chronic ethanol binge treated mice. The chronic ethanol feeding (four weeks) and three binge treatment was performed as described under “Experimental Section”. The hepatic nuclear extracts were used for western blotting followed by quantitative imaging [30]. Images of representative blots are shown. Values are mean ± SE (*n* = 4 mice). a: significant compared to control (*p* < 0.05); b: significant from chronic ethanol group (*p* < 0.05); c: significant compared to control-binge. C: Control (pair fed); E: Chronic ethanol; CB: Control ethanol binge; EB: Chronic-ethanol-binge. (**A**) H3PS10; (**B**) H3PS28.

Post-translational modifications in histone proteins by ethanol have been shown earlier [14,15]. However, the acute on chronic ethanol influence on these modifications in mouse liver is not known and was therefore monitored. Increased phosphorylation of histone H3S10 (Figure 3A) and H3S28 (Figure 3B) indicate chromatin remodeling and gene transcription [14,15,16,17,23]. Phosphorylation of histone H3S10 (Figure 3A) and H3S28 (Figure 3B) did not change after chronic ethanol or binge administration alone whereas chronic ethanol followed by binge caused a significant increase in histone H3S10 and histone H3S28 phosphorylation (Figure 3A,B).

### 2.3. Levels of Dimethylated H3 K4, Dimethylated H3 K9, and Trimethylated H3K9

**Figure 4:**
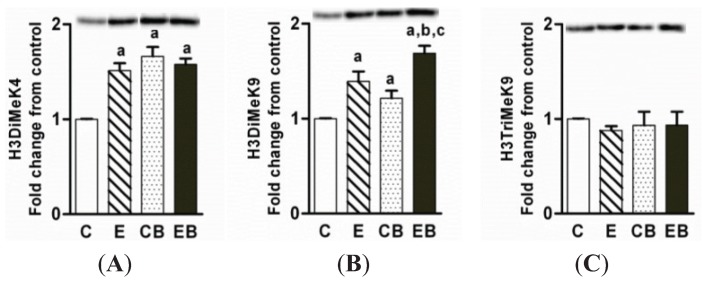
Levels of dimethylated H3K4, dimethylated histone H3K9, and trimethylated histone H3K9 in chronic ethanol and binge treated animals. The experimental protocol was as detailed in the “Experimental Section”. The representative western blot images are also shown above the histograms. Values are mean ± SE (*n* = 4 mice). a: significant compared to control (*p* < 0.05); b: significant from chronic ethanol group (*p* < 0.05); c: significant compared to control-binge. C: Control (pair fed); E: Chronic ethanol; CB: Control ethanol binge; EB: Chronic-ethanol-binge. (**A**) H3DiMeK4; (**B**) H3DiMeK9; (**C**) H3TriMeK9.

Histone H3K4 methylation is implicated in transcriptional activation whereas histone H3K9 dimethylation and H3K9 trimethylation are involved in silencing of gene expression [15,17,18,25]. H3K4 dimethylation increased to similar levels in chronic ethanol, binge ethanol, and chronic ethanol-binge groups (Figure 4A). H3K9 dimethylation also increased in chronic ethanol, binge, and chronic ethanol binge group, but the extent of histone H3K9 dimethylation was more marked in chronic ethanol-binge group (Figure 4B). In contrast to above changes, the levels of trimethylated H3K9 remained unaltered in all the three groups (Figure 4C).

### 2.4. Increased H3K9 Acetylation, H3K9 Acetylation/S10 Phosphorylation and H2AX Phosphorylation

**Figure 5:**
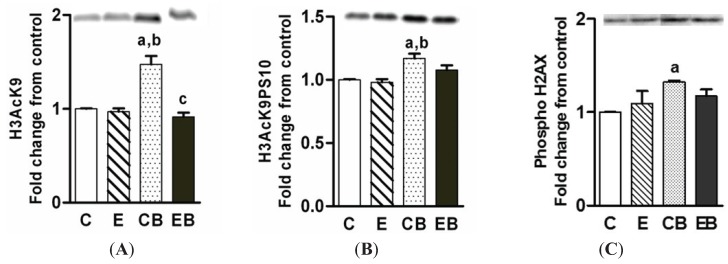
Levels of H3AcK9 (**A**); H3AcK9PS10 (**B**); and phospho-H2AX (**C**). The experimental details were as described in the “Experimental Section”. The nuclear levels of these components were determined by western blotting (representative images are shown above the histograms). Values are mean ± SE (*n* = 4 mice). a: significant compared to control (*p* < 0.05); b: significant from chronic ethanol group (*p* < 0.05). C: Control (pair fed); E: Chronic ethanol; CB: Control ethanol binge; EB: Chronic-ethanol binge.

Acetylation of histone H3K9 is one the significant epigenetic effects of ethanol *in vitro* and *in vivo* under the setting of binge and chronic ethanol binge in rats [15,30]. H3AcK9 levels did not increase after chronic ethanol intake (Figure 5A), increased after binge ethanol alone, and was not altered after chronic ethanol binge (Figure 5A). Histone H3K9 acetylation is often associated with accumulation of histone H3 dually modified by H3K9 acetylation/S10 phosphorylation (H3AcK9PS10) [15,22,23]. The levels of H3AcK9PS10 did not change under these treatment conditions except for a small increase in binge alone (Figure 5B). Histone H2AX phosphorylation is implicated in nuclear DNA repair and is modulated by histone H3 acetylation [33,34,35]. Therefore, we examined H2AX phosphorylation in ethanol treated mice groups. H2AX phosphorylation showed a pattern similar to H3K9 acetylation with a significant increase in the acute/binge group (Figure 5C).

### 2.5. Increased Nuclear Levels of GCN5, HDAC3 and Decreased Levels of Phospho-CREB in Chronic Ethanol, Binge and Chronic Ethanol-Binge Groups

**Figure 6:**
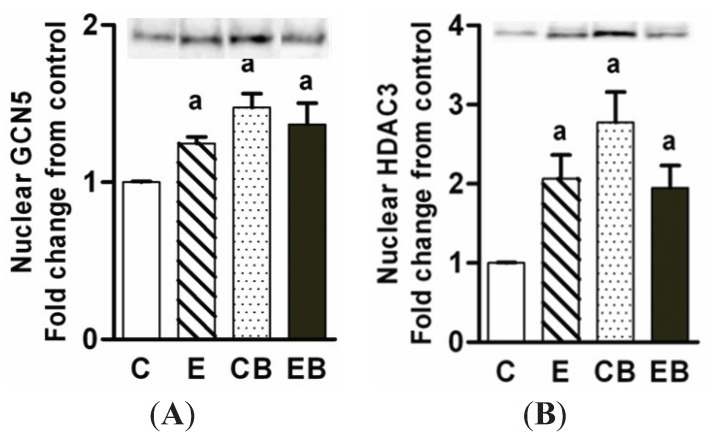
Levels of GCN5 (**A**); and HDAC 3 (**B**) in chronic ethanol-binge treated mice. The experimental protocol were as in the “Experimental Section”. The levels of GCN5 and HDAC3 were determined by western blotting and the representative images of blots are shown above the histograms. Values are mean ± SE (*n* = 4 mice). a: significant compared to control (*p* < 0.05). C: Control (pair fed); E: Chronic ethanol; CB: Control ethanol binge; EB: Chronic-ethanol-binge.

Increased expression of HAT GCN5 and increased H3K9 acetylation by ethanol occurs in hepatoma cells [36]. Therefore we determined GCN5 protein levels in the liver nuclear extracts. GCN5 levels increased in chronic ethanol, binge, and chronic ethanol binge groups (Figure 6A) although H3K9 acetylation was significantly increased only in binge group (Figure 5A). It is not known if the differential pattern of GCN5 expression and H3K9 acetylation are due to altered levels of histone deacetylases. We therefore determined HDAC3 levels since HDAC3 levels are increased in acute ethanol administration in mice [21]. The HDAC3 protein levels in the nuclear extracts were increased in all three groups with maximum increase in the binge group but this increase was not statistically different from other groups (Figure 6B).

Phosphorylated CREB (cyclic AMP response element binding protein) has been shown to stimulate CBP/p300 mediated acetylation of H3K9 and is one of the important transcriptional regulator of hepatic metabolic and inflammatory responses [35,37,38].

**Figure 7:**
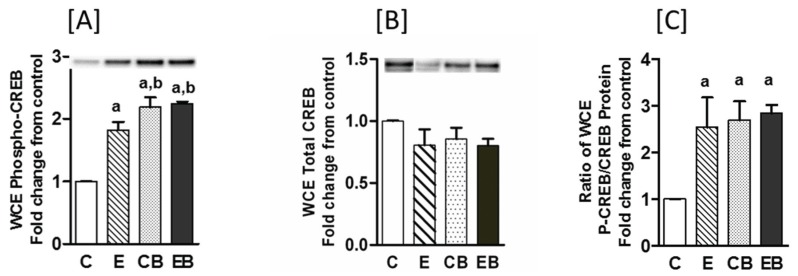

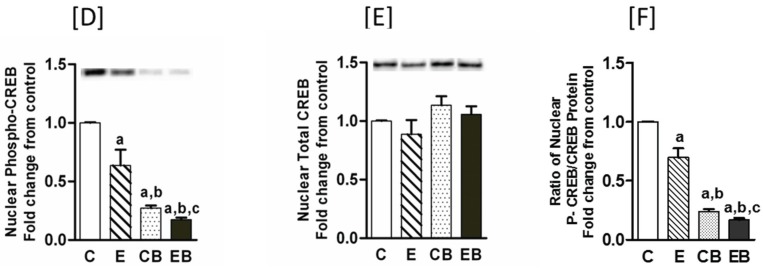
Phosphorylated CREB and CREB protein in hepatic extracts from chronic ethanol-binge treated mice. Changes in CREB and P-CREB were determined by western blotting of whole cells extracts (WCE, upper panel (**A**–**C**)); and nuclear fraction (lower panel (**D**–**F**)). Images of representative blots are also shown. The ratio of P-CREB/CREB are also shown. Values are mean ± SE (*n* = 4 mice). a: significant compared to control (*p* < 0.05); b: significant from chronic ethanol group (*p* < 0.05); c: significant compared to control-binge. C: Control (pair fed); E: Chronic ethanol; CB: Control ethanol binge; EB: Chronic-ethanol-binge.

Phosphorylation of CREB that usually occurs in the cytosol facilitates its translocation into the nucleus [37,38]. Therefore, we determined the levels of phosphorylated CREB (P-CREB) and CREB protein in the whole cell (Figure 7A–C) and nuclear extracts (Figure 7D–F). In the whole cell extracts P-CREB increased in all the groups (Figure 7A) but the total CREB protein did not change in either of the groups (Figure 7B). However, there was a modest but statistically significant increase in P-CREB in binge (CB) and chronic ethanol-binge (EB) groups when compared to chronic ethanol (E) group (Figure 7A). The ratio of P-CREB/CREB was similar in all the three groups (Figure 7C). In contrast, in the nuclear fraction the levels of P-CREB gradually decreased in the following order: chronic > binge alone > chronic-binge (Figure 7D). The decrease in the binge group was more marked compared to chronic ethanol group but the most significant decrease occurred in the chronic ethanol-binge group. However, nuclear levels of CREB protein was not significantly different among the groups (Figure 7E) and changes in the ratio of P-CREB/CREB paralleled changes in nuclear levels of P-CREB (Figure 7F).

## 3. Discussion

Heavy binge drinking in patients who are chronically consuming alcohol is the most common cause for the worsening of liver damage in chronic alcoholic liver disease [7,8,9]. To mimic this scenario we have developed an experimental animal model of chronic ethanol followed by repeat binge episode to examine augmentation of liver injury in mice. Earlier, we reported a rat model for chronic ethanol followed by three doses of acute binge administration [29,30]. In the rat model it was shown that 3 binge is more damaging than single binge in chronically treated animals [29]. In the present study, we have used a mouse model because both rat and mouse models serve as good experimental models to study alcoholic liver disease [32]. Moreover, the mouse model is useful for studying the progression of ALD due to increased susceptibility of mice for alcoholic liver injury and the feasibility of genetic manipulation in mice [1,31,32]. In this study, we explored for the first time, dysregulated methionine metabolism, adenosine accumulation and epigenetic histone modifications and enzymes related to histone modifications as determinates of augmentation of liver injury after binge ethanol administration in chronically ethanol treated mice. Although the pattern of injury was comparable to the rat model, there were differences in the magnitude of changes in biomarkers of liver injury. Additionally, the patterns of changes in histone modifications were distinct for the rat and mice models. Increased steatosis and elevated serum ALT levels were observed in the chronic ethanol binge group in this study and suggest exacerbation of chronic ethanol induced injury by binge administration. Ethanol causes increased levels of CYP2E1 *in vivo* and in primary hepatocytes thus contributing to ethanol induced oxidative stress [10,11,33]. Although CYP2E1 levels are increased after binge and chronic ethanol, the increase is most marked after chronic ethanol-binge suggesting an important role for oxidative stress in enhancing liver injury by acute on chronic ethanol ingestion. The role of CYP2E1 levels in the pathogenesis is underscored by the prevention of binge and chronic ethanol induced liver injury in CYP2E1 knock out mice [10,31,32]. Hepatic glutathione levels have been reported to be elevated, decreased, or unaltered by ethanol feeding [39,40]. In the present study, we observed that glutathione levels in whole liver extracts were not altered. In fact, the levels were mildly elevated in all three groups which could be a protective response of the liver to ethanol exposure.

Enhanced adenosine release from hepatic slices from alcohol treated mice and increased expression of adenosine receptors have been reported in animal models of alcoholic liver injury and in humans [12,13]. Moreover, adenosine receptor antagonists ameliorate liver injury in chronic alcohol treated mice [12,13]. The levels of hepatic adenosine were lower (compared to control) after chronic ethanol intake and remained unaltered after binge. However, its levels roughly doubled above control in chronic ethanol-binge liver. Therefore, increased levels of adenosine may contribute to the potentiation of chronic alcoholic liver injury by binge.

Dysregulation of methionine metabolism is one of the hepatotoxic effects of ethanol [11]. Hepatic *s*-adenosyl methionine (SAM) levels increased after chronic ethanol but decreased by chronic ethanol binge. Although increased SAM levels indicate adaptive response to chronic ethanol administration, the significant decrease in SAM levels and decreased ratio of SAM/SAH suggest increased cytotoxic effects of chronic ethanol-binge combination under the experimental protocols utilized in the present study. Decreases in SAM levels are associated with impairment in essential methylation reactions through inhibition of various methyltransferases including phosphatidylethanolamine methyltransferase, isoprenylcysteine carboxyl methyltransferase and protein L-isoaspartate methyltransferase. Decreased activity of these enzymes is associated with increased steatosis, increased apoptosis and increased accumulation of damaged proteins seen in alcoholic liver disease [11,40].

Chronic ethanol intake or binge alone caused insignificant increase in histone H3S10 and H3S28 phosphorylation whereas binge after chronic ethanol increased histone H3S10 and H3S28 phosphorylation. In contrast to histone phosphorylation, histone H3K4 and H3K9 dimethylation increased in chronic, binge, and chronic binge groups. H3K4 dimethylation is usually associated with induction of gene expression and H3K9 dimethylation is associated with silencing of gene expression [15]. This suggests different sets of modified histone residues in chromatin remodeling complex with simultaneous upregulation and down regulation of different genes [18]. In this chronic-repeat binge mice model histone H3K9 trimethylation was not affected by ethanol. Moreover, increase in histone H3K4 and H3K9 dimethylation accompanied a decrease in SAM in chronic ethanol binge group suggesting histone H3 dimethylation by ethanol may occur independent of the availability of methyl donor and may involve other mechanisms. The cytokine tumor necrosis factor (TNFα) and transcription factor early growth response gene (egr-1) are upregulated by histone H3 phosphorylation, and TNFα is silenced by histone H3K9 methylation [23,41,42]. Therefore, an increase in histone H3 phosphorylation in chronic ethanol-binge but not in chronic ethanol or binge suggests additional epigenetic modifications contributing to the amplification of liver injury. It is noteworthy that the changes in global histone modification are further fine-tuned by gene specific histone modifications resulting in multiple effects on gene expression as seen in chronic ethanol treated rats with unaltered global histone H3K9 acetylation but increased recruitment of histone H3K9 to the ADH1 promoter but not to c-jun, although expression of mRNA for both genes were increased [26].

In contrast to H3S10 and H3S28 phosphorylation, H3K9 acetylation was increased in the binge ethanol but not in the chronic ethanol-binge group. Histone H3K9 acetylation is dependent on a balance between the activation of histone acetyl transferases and histone deacetylases. We have reported a role of GCN5 in ethanol induced H3K9 acetylation in ethanol metabolizing human hepatoma cell line (VA13 cells) [36]. Levels of GCN5 increased in all the three groups although H3K9 acetylation occurred only after binge. HDAC3 levels also increased in all three groups. This may indicate a delicate balance between HAT and HDAC that is modulated by ethanol exposure. Dually modified H3AcK9/PS10 also increased by binge and suggests acetylation of phosphorylated histone H3. In this regard, HATs such as ATF2 histone acetyl transferase show preference for phosphorylated histones. Increased H3K9 acetylation is also accompanied by increased histone H2AX phosphorylation by binge. Role for GCN5 histone acetyl transferase in H3K9 acetylation associated with H2AX phosphorylation by SW1/SNF chromatin complex has been recently reported [34]. H2AX phosphorylation has been implicated in DNA repair [33,34]. However, this protective response is compromised when binge was administered to chronic ethanol treated mice. It is relevant to note that chronic ethanol binge also resulted in decreased levels of H2AX phosphorylation in rats [33].

CREB is a key transcription factor playing a pivotal role in liver regeneration, hepatic carbohydrate metabolism, suppression of hepatic lipogenesis, and hepatic inflammatory responses. The levels of phospho-CREB in the whole cell extract were significantly increased in chronic ethanol, binge and chronic ethanol binge but nuclear levels were markedly decreased with most pronounced decrease noted in the ethanol binge group. Thus, dysregulation of nuclear P-CREB levels may be an underlying mechanism in alcohol induced liver injury by both binge and chronic ethanol intake. One mechanism that may contribute to transcriptional regulation is CREB dependent CBP/p300 mediated histone acetylation [35,37].

## 4. Experimental Section

### 4.1. Reagents

Antibodies to histone H3AcK9, phospho-H3 ser 10, phospho-H3 ser 10, phospho-H3 ser 28, histone H3 protein, dimethyl H3-lys4, dimethyl H3-lys9, phopsho-H2AX were obtained from Chemicon (Temecula, CA, USA). Antibodies to GCN5 and CYP2E1 were procured form Santa Cruz (Dallas, TX, USA). Antibody to HDAC3, phospho-CREB, and CREB protein were procured from Cell signaling (Danvers, MA, USA). Triglyceride assay kit (Cat #F6428), protease inhibitors cocktail (p8340), and anti β-actin antibody were purchased from Sigma-Aldrich (St. Louis, MO, USA).

### 4.2. Experimental Design for Chronic Ethanol Feeding and Binge Administration

Seven week old C57BL/6J male mice, were purchased from Harlan (Indianapolis, IN, USA) and housed under a 12-h/12-h light/dark cycle. Animals were permitted ad libitum consumption of standard laboratory chow for one week to acclimatize. This was followed by feeding animals with Lieber-DeCarli liquid diet (Dyets, Inc., Bethlehem, PA, USA; [29]). Ethanol was progressively introduced into the liquid diet starting at 1% (wt/vol) for 2 days, increased to 2% for 2 days, 3% for 2 days, and 4% from day 7 onwards for 4 weeks. Pair-fed control mice were given a liquid diet in which ethanol was replaced by dextrin/maltose to maintain the isocaloric intake in the two groups. At the end of 4 weeks of feeding, mice were divided into four groups: Control group (C) received water, chronic ethanol group (E) received water, control-binge group (CB) received three doses of ethanol, and chronic ethanol-binge group (EB) received three doses of ethanol. The three doses of binge were administered intragastrically (3.5 gm/kg body weight) after diluting ethanol to 32% (*v*/*v*) in sterile water and orally gavaged to the stomach using a 20-gauge stainless steel blunt tipped needle. The average amount of intragastric alcohol solution was about 0.45 mL. The three ethanol binge were given consecutively each 12 h apart. Four hours after the last binge ethanol administration blood and liver samples were collected. Liver was perfused with cold phosphate buffered saline containing phosphatase inhibitors before collection. A small portion of the liver was placed in formalin for immunohistochemistry and the reminder was frozen in liquid nitrogen and stored at −70 °C. The animal care and protocol used in this study was approved by the University of Missouri Animal Care Committee (Protocol #8175) and complied with NIH guide for the care and use of laboratory animals.

### 4.3. Biochemical and Histological Studies

Serum ALT levels were measured by kinetic ALT assay in an automated analyzer (University of Missouri Research Animal Diagnostic Laboratory, Columbia, MO, USA). The levels of Liver SAM, SAH, adenosine and GSH were measured by high performance liquid chromatography as described earlier [11,30]. Formalin fixed liver tissue was embedded in paraffin, sectioned and stained with hematoxylin and eosin (H & E). Liver triglyceride levels were determined essentially as reported earlier by us [29]. Thirty mg of liver tissue was homogenized in 0.5 mL hypotonic buffer containing 20 mM Tris, 2% Triton X-100, and Sigma protease inhibitor cocktail (p8340) in a 2 mL capacity Dounce homogenizer with pestle A. The sample was heated to 60 °C followed by centrifugation at 13,000× *g* for 5 min. The supernatant was used for triglyceride estimation using the assay kit and protocol provided by the supplier (Cat #F6428, Sigma-Aldrich Company, St. Louis, MO, USA).

### 4.4. Nuclear Isolation and Immunoblot Analysis

The whole cell extract and nuclear extracts were prepared at 4 °C as described earlier [30]. After homogenizing the frozen liver tissue in lysis buffer (containing 50 mM Tris. HCl. pH 7.4, 25 mM KCl, 5 mM MgCl_2_, 5 mM glycerophosphate, 1 mM EDTA, 1 mM Na-orthovanadate, 1 mM EGTA, 1 mM DTT, and Sigma protease inhibitor cocktail (p8340)), a small portion of the whole cell extract was saved. The remainder of the homogenate was layered over 1.0 M sucrose cushion. After centrifugation at 1600× *g* for 10 min at 4 °C, the cytosolic fraction (supernatant) was transferred to a precooled microcentrifuge tube, frozen in liquid nitrogen and stored at −70 °C. The nuclear pellet was further suspended in 1.0 M sucrose containing 0.25% NP-40 and passed through a 26-gauge needle 6 times and centrifuged at 1600× *g* for 10 min. This step removed contaminated endoplasmic reticulum and plasma membrane. The nuclear pellet was again re-suspended in 1.0 M sucrose and centrifuged at 1600× *g* for 10 min followed by washing the pellet once with homogenization buffer. The purity of the nuclear fraction was verified by the absence of b-tubulin and calreticulin and presence of histone H3 [30]. The nuclear extract was prepared in high salt detergent buffer (0.5 M NaCl, 1% Triton X-100, 1% deoxycholate and 0.1% SDS). Bio-Rad DC protein assay was used to determine the protein concentrations in whole cell extract and nuclear extract.

The whole liver lysate protein or nuclear fraction (40 µg) was separated by 10% or 15% SDS-PAGE followed transfer onto nitrocellulose membrane using Bio-Rad Trans-Blot apparatus. Membrane was washed with 20 mM Tris, pH 7.5, containing 0.1% Tween 20 and 150 mM NaCl (TBST) and blocked with TBST containing 5% nonfat dry milk for 2 h at room temperature. Membrane was incubated with primary antibody overnight at 4 °C in blocking buffer and washed with TBST, followed by incubation with corresponding secondary antibody conjugated with horseradish peroxidase for 1 h at room temperature. Immunoblots were visualized using chemiluminescent reagent (Pierce Chemicals, Rockford, IL, USA). LSA-3000 imaging system (Fujifilm Life Sciences, Stamford, Connecticut, USA) was used to capture chemiluminescence and Multi Gauge™ software (Fujifilm Life Sciences) was used for quantitation of immune blots. The chemiluminescence intensity was within the linear range of detection. Beta actin and histone H3 protein immune blots were used for equal protein loading of whole cell extracts and nuclear extracts respectively. Repeat immunoblotting was done by stripping the membrane using Restore Western blot stripping buffer (Pierce).

### 4.5. Data Analysis

Data obtained from 4 animals in each group were used for analysis. The data are expressed as mean ± S.E.M. ANOVA was used for statistical analysis and differences with a *p*-value of <0.05 were considered statistically significant.

## 5. Conclusions

In conclusion, augmentation of liver injury by repeat acute binge in chronic ethanol treated mice is accompanied by increased levels of CYP2E1 protein, ALT, triglyceride, decrease in the SAM:SAH ratio, marked increase in hepatic adenosine levels, and increased H3 phosphorylation , a histone modification known to enhance TNFα gene expression. The association between gene polymorphism for CYP2E1, methionine metabolism, and TNFα has been considered in the alcoholic liver injury in humans [43]. Analogous changes observed in our acute on chronic model suggest that it may be a relevant experimental model for the study of progression of alcoholic liver injury. On the other hand, the increase in H2AX phosphorylation in binge but its suppression observed in the chronic ethanol binge group suggests that chronic ethanol intake antagonize the protective adaptive response of ethanol binge. The distinct pattern of hepatic methionine metabolism and epigenetic histone modifications but similar pattern of CYP2E1 protein induction in rat [30,33] and mouse (this study) models of ALD highlight common and distinct mechanisms of pathogenesis of liver damage. It may be tempting to suggest that the use of acute repeat binge on chronic ethanol intake for extended period of time may enhance alcoholic liver injury further mimicking the progression of liver injury in a setting commonly seen in humans.
